# A nonlinear fractional epidemic model for the Marburg virus transmission with public health education

**DOI:** 10.1038/s41598-023-46127-7

**Published:** 2023-11-07

**Authors:** Emmanuel Addai, Adejimi Adeniji, Mercy Ngungu, Godfred Kuffuor Tawiah, Edmore Marinda, Joshua Kiddy K. Asamoah, Muhammad Altaf Khan

**Affiliations:** 1 College of Computer and Information Science, University of Arkansas at Little Rock, Little Rock, Arkansas State 72204 USA; 2 Department of Mathematics and Statistics, Tshwane University of Technology, Pretoria, South Africa; 3 Human Sciences Research Council (HSRC), Pretoria, South Africa; 4 College of Biomedical Engineering, Taiyuan University of Technology, Shanxi Taiyuan, 030024 China; 5 Department of Mathematics, Kwame Nkrumah University of Science and Technology, Kumasi, Ghana; 6 Faculty of Natural and Agricultural Sciences, University of the Free State, Bloemfontein, South Africa

**Keywords:** Applied mathematics, Diseases, Infectious diseases

## Abstract

In this study, a deterministic model for the dynamics of Marburg virus transmission that incorporates the impact of public health education is being formulated and analyzed. The Caputo fractional-order derivative is used to extend the traditional integer model to a fractional-based model. The model’s positivity and boundedness are also under investigation. We obtain the basic reproduction number $\mathfrak {R_0}$ and establish the conditions for the local and global asymptotic stability for the disease-free equilibrium of the model. Under the Caputo fractional-order derivative, we establish the existence-uniqueness theory using the Banach contraction mapping principle for the solution of the proposed model. We use functional techniques to demonstrate the proposed model’s stability under the Ulam-Hyers condition. The numerical solutions are being determined through the Predictor-Corrector scheme. Awareness, as a form of education that lowers the risk of danger, is reducing susceptibility and the risk of infection. We employ numerical simulations to showcase the variety of realistic parameter values that support the argument that human awareness, as a form of education, considerably lowers susceptibility and the risk of infection.

## Introduction

Marburg virus is a highly infectious virus that belongs to the Filoviridae family, which also includes the Ebola virus. The virus was first identified in 1967, during simultaneous outbreaks in Marburg, Germany^1^. These outbreaks were associated with laboratory work involving African green monkeys imported from Uganda. Since then, sporadic outbreaks of Marburg virus disease have occurred in various countries in Africa, including Uganda, Angola, and Kenya^1–4^. The natural reservoir for Marburg virus is believed to be fruit bats of the Pteropodidae family, which are found throughout sub-Saharan Africa. The virus is transmitted to humans through direct contact with the bodily fluids of infected individuals or animals^5^. The incubation period for the virus ranges from 2 to 21 days, with symptoms typically appearing between 5 and 10 days after infection^6^. Marburg virus causes severe and often fatal hemorrhagic fever in humans and nonhuman primates. The virus attacks multiple organs and systems in the body, leading to a range of symptoms, including fever, headache, muscle pain, vomiting, and diarrhea. In severe cases, the virus can cause hemorrhagic fever, which is characterized by bleeding from the eyes, ears, and mouth, as well as internal bleeding. The case fatality rate for Marburg virus disease ranges from 24% to 88%, depending on the outbreak and the quality of care available^1,7^. The impact of Marburg virus on the African continent has been significant. Given the severity of Marburg virus disease and the potential for outbreaks to cause significant harm, it is crucial that public health officials remain vigilant and prepared to respond to any new cases or outbreaks. The recent Marburg virus infection in sub-Saharan African countries, namely Equatorial Guinea, Tanzania, Ghana, Guinea, and Uganda has been considered to be significant which called for World Health Organization’s (WHO) in-depth epidemiological investigation, intervention and potential treatment^8,9^.

It was widely recognized that the social behaviour of individuals within a society has had a significant impact on the occurrence of any epidemic. It was noted that behaviour modification was crucial in the spread of disease. It was noted that behavioural change played a very important role in the spread of the disease^10,11^. When an infectious disease first appears, the first priority is to rapidly inform the public about the illness and its protective measures. Although widespread vaccination is the most effective method of disease management, vaccination is expensive and sometimes only temporarily confers immunity. In developing and underdeveloped countries, public health educational initiatives on Marburg virus infection are essential for managing and preventing the disease and can raise people’s health literacy and awareness of the virus. The awareness campaigns not only educate the public about the Marburg virus, but also recommend important preventive measures like the use of face masks, improved hygiene, and avoidance of congregated areas, among others, which can sometimes alter people’s behaviour and lower the risk of infection.

While dealing with an epidemic, it is crucial to anticipate future events, understand how to stop the virus from spreading, and provide the required instructions so that the situation may be handled before it gets out of hand. The forecasting, investigation, and creation of significant strategies to battle infectious diseases have been made possible by numerous researchers from a broad range of disciplines. Several scientists have used mathematical modelling to understand the intricacies of how viruses spread^12–14^. In a broad range of systems and processes, fractional derivatives are an effective tool for explaining memory and hereditary characteristics^15–17^. With fractional-order modelling, we have such an additional variable (order of the derivative) that is helpful for numerical techniques. The dynamics of many disease transmission have been investigated using fractional-order modelling. For instance, mathematical model for HIV/AIDS^18^, Ebola-malaria co-infection model^19^, smoke age-specific model^20^, monkeypox transmission dynamics^21–23^ Middle East Lungs Coronavirus dynamism transmission model^24^, COVID-19 model^25,26^, Hepatitis E disease model^27^ are all examples of fractional model used in the past. For more application on fractional derivatives see, for instance^28,29^, and references therein. The Caputo fractional order derivative is very helpful for discussing actual situations since it enables the inclusion of conventional starting and boundary conditions in the derivation and because the derivative of a constant is zero, unlike the Riemann-Liouville fractional derivative.

Inspired by the advantages of Caputo derivative that gives standard beginning and steady state in the derivation over other fractional derivatives which have been used in other research^30–32^ areas of infectious diseases, this work consider Caputo fractional model to discuss how various variables affect the mathematical simulation of the dynamics of Marburg virus transmission incorporating public health education. It is important to note that this is relatively the first time a fractional order model has been presented to simulate the aforemention topic. The following highlight the innovative nature of our study:

i. A novel fractional model defined in the Caputo sense is used to examine the dynamics of Marburg virus transmission incorporating the impact of public health education.
ii. The reproduction number $\mathfrak {R_0}$ for the proposed model is being derived along with the disease free equilibrium points for the system.
iii. We demonstrate the existence and uniqueness solutions of the dynamics of Marburg virus transmission incorporating the impact of public health education by employing the Banach contraction mapping principle.
iv. The fractional controlling system of equations underwent a stability study using the Hyers-Ulam-type stability criteria.
v. To validate the theoretical components and conclusions of the suggested model, an effective numerical technique is adopted.
vi. The obtained results demonstrate the effectiveness of the suggested model in providing some new insights into the dynamics of the Marburg virus infection as well as some preventative actions.

The paper is structured in the manner described below. In Section "Preliminaries", the basic definitions and lemmas are presented. The formulation of the Marburg virus infection model based on the system of a deterministic mathematical model and the Caputo fractional derivative in Section "Model formulation". Section "Basic qualitative properties of the model" deals with equilibrium points and the basic reproduction number. The mathematical analysis of the existence-uniqueness of our suggested Marburg virus transmission model is covered in Section "Existence and uniqueness". In Section "HU stability", the stability results of the Marburg virus transmission model is shown and discussed. Sections "Numerical simulation" and "Numerical simulation and discussion" deal with the numerical framework and simulations, respectively. Section "Conclusion" of the paper concludes the work.

## Preliminaries

n this section, we recall some critical concepts, lemmas, and definitions to study our proposed model.

### Definition 2.1

^33^ The Caputo fractional derivative of order $\gamma$ ($\gamma >0$) of ${\mathfrak {u}}$ is given by

$$\begin{aligned} {}^{{\mathfrak {c}}}{\mathcal {D}}^{\gamma }{\mathfrak {u}}(x)=\frac{\int ^{x}_{a}(x-y)^{n-\gamma -1}{\mathfrak {u}}^{(n)}(y)\textrm{d}y}{\Gamma (n-\gamma )}. \end{aligned}$$

The Riemann-Liouville fractional integral of $\gamma$ order of ${\mathfrak {u}}$ is given by

$$\begin{aligned} {\mathcal {I}}^{\gamma }_{0^{+}}{\mathfrak {u}}(x)=\frac{\int ^{x}_{0}(x-y)^{\gamma -1}{\mathfrak {u}}(y)\textrm{d}y}{\Gamma (\gamma )}, \end{aligned}$$

where $n=[\gamma ]+1$, $[\gamma ]$ denotes the integer part of number $\gamma$, provided that the right side is pointwise defined on (0, 1).

### Lemma 2.1

^34^ Assume that ${\mathfrak {u}}\in C[0,+\infty ) \cap L[0,+\infty )$ with the derivative of order *n*, then

$$\begin{aligned} {\mathcal {I}}^{\gamma }_{0^{+}}~ ^{c}{\mathcal {D}}^{\gamma }_{0^{+}}{\mathfrak {u}}(t) = {\mathfrak {u}}(t) +k_{ 1} + k_{2} t +k_{ 3} t^{ 2} + \cdot \cdot \cdot + k_{ n} t^{n-1}, ~\gamma > 0 \end{aligned}$$

where $$k_{ i}\in R, i = 1,2,\cdot \cdot \cdot ,n$$ and $$n = [\gamma ]+1.$$

## Model formulation

In this section, we present the formulation of the model, which we will be studying in this paper. For this, we have considered only human population and divided it into seven different compartments, which are susceptible humans without public health education about the Marburg virus transmission (at a time) *S*(*t*); susceptible humans with public health education *P*(*t*); Exposed individuals ${\mathcal {E}}$; Undetected infected individuals ${\mathcal {I}}_{u}$; Detected Marburg virus infection ${\mathcal {I}}_{d}$; Hospitalisation of Marburg virus infected individuals ${\mathcal {H}}$; Recovery from Marburg virus infection ${\mathcal {R}}$. We assume that, at any time, the educated susceptible group may act as ignorantly and enter the class of susceptible at a constant rate $\xi$. The total population as a whole is provided by

$$\begin{aligned} N(t)={\mathcal {S}}(t)+{\mathcal {P}}(t)+{\mathcal {E}}(t)+{\mathcal {I}}_{u}(t)+{\mathcal {I}}_{d}(t)+{\mathcal {H}}(t)+{\mathcal {R}}(t). \end{aligned}$$

Susceptible humans enter the population, either through birth or migration, at a rate $\Pi$ and are infected with the Marburg virus at rate $\lambda$, where

$$\begin{aligned} \lambda =\frac{\beta (\gamma _{1}{I}_{u}+ {I}_{d}+ \gamma _{2}H)}{N(t)}. \end{aligned}$$

Here, the $\beta$ is the transmission rate. $\gamma _{1}$ and $\gamma _{2}$ are the modification parameters of ${I}_{u}$ and *H*, respectively. Considering the interrelationship, the infection model used in analyzing the dynamics of Marburg virus transmission incorporating the impact of public health education is given by the following deterministic system of nonlinear differential equations

1 $$\begin{aligned} \left\{ \begin{array}{llllllllll} \frac{dS(t)}{dt}=\Pi + \xi P(t)-(\lambda +\mu +\phi )S(t), \\ \frac{dP(t)}{dt}=\phi S(t)-(\nu \lambda +\mu +\xi )P(t), \\ \frac{dE(t)}{dt}=\lambda S(t) + \nu \lambda P(t)-(r + \mu ) E(t), \\ \frac{dI_{u}(t)}{dt}=r(1-\varepsilon )E(t)-(\eta _{1}+\psi +\mu +\delta )I_{u}(t),\\ \frac{dI_{d}(t)}{dt}=r\varepsilon E(t)+\psi I_{u}(t)-(\vartheta +\mu +\delta )I_{d}(t), \\ \frac{dH (t)}{dt}=\vartheta I_{d}(t)- (\eta _{2}+\delta + \mu )H(t), \\ \frac{dR (t)}{dt}=\eta _{1}I_{u}+\eta _{2}H(t)-\mu R(t),\\ \end{array} \right. \end{aligned}$$

**Figure 1:**
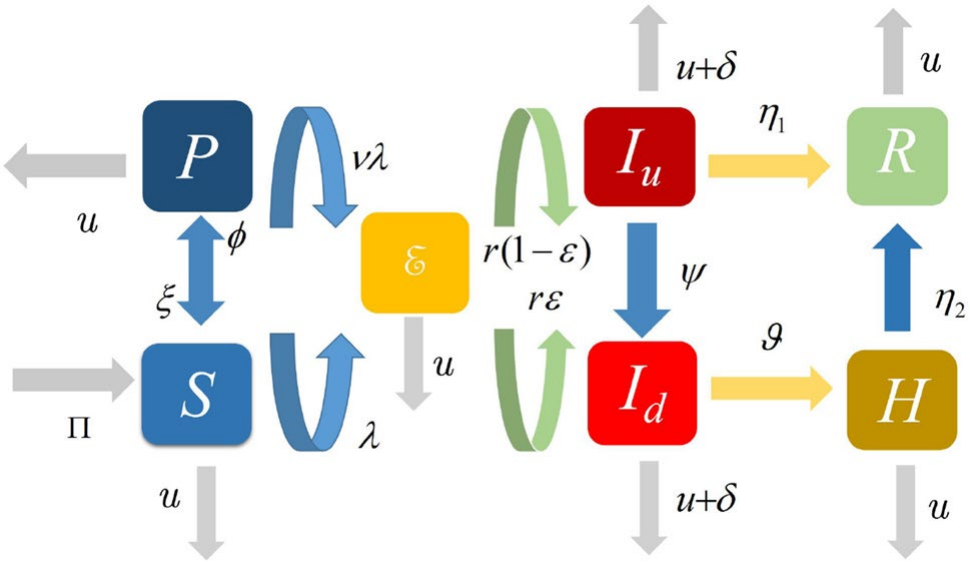
Transfer diagram for the Marburg virus transmission.

The flow diagram of the model is presented in Fig. 1 while the description of the rest of parameters are presented in Table 1. According to the explanation of time-dependent kernel defined by the power law correlation function, presented in Ref.^35^, our considered Caputo fractional order derivative model for the dynamics of Marburg virus transmission is defined as follows;

2 $$\begin{aligned} \left\{ \begin{array}{llllllllll} {}^{{\mathfrak {c}}}{\mathcal {D}}^{\gamma }{\mathcal {S}}(t) =\Pi + \xi {\mathcal {P}}(t)-(\lambda +\mu +\phi ){\mathcal {S}}(t), \\ {}^{{\mathfrak {c}}}{\mathcal {D}}^{\gamma }{\mathcal {P}}(t) =\phi {\mathcal {S}}(t)-(\nu \lambda +\mu +\xi ){\mathcal {P}}(t), \\ {}^{{\mathfrak {c}}}{\mathcal {D}}^{\gamma }{\mathcal {E}}(t) =\lambda {\mathcal {S}}(t) + \nu \lambda P(t)-(r + \mu ) {\mathcal {E}}(t), \\ {}^{{\mathfrak {c}}}{\mathcal {D}}^{\gamma }{\mathcal {I}}_{u}(t) =r(1-\varepsilon ){\mathcal {E}}(t)-(\eta _{1}+\psi +\mu +\delta ){\mathcal {I}}_{u}(t),\\ {}^{{\mathfrak {c}}}{\mathcal {D}}^{\gamma }{\mathcal {I}}_{d}(t) =r\varepsilon {\mathcal {E}}(t)+\psi I_{u}(t)-(\vartheta +\mu +\delta ){\mathcal {I}}_{d}(t), \\ {}^{{\mathfrak {c}}}{\mathcal {D}}^{\gamma }{\mathcal {H}}(t) =\vartheta {\mathcal {I}}_{d}(t)- (\eta _{2}+\delta + \mu ){\mathcal {H}}(t), \\ {}^{{\mathfrak {c}}}{\mathcal {D}}^{\gamma }{\mathcal {R}} (t) =\eta _{1}{\mathcal {I}}_{u}+\eta _{2}{\mathcal {H}}(t)-\mu {\mathcal {R}}(t),\\ \end{array} \right. \end{aligned}$$

with the initial conditions; ${\mathcal {S}}(0)=l_{1}\ge 0,{\mathcal {P}}(0)=l_{2}\ge 0,{\mathcal {E}}(0)=l_{3}\ge 0$,${\mathcal {I}}_{u}(0)=l_{4}\ge 0,$ ${\mathcal {I}}_{d}(0)=l_{5}\ge 0,{\mathcal {H}}(0)=l_{6}\ge 0,{\mathcal {R}}(0)=l_{7}\ge 0.$ Where $^{{\mathfrak {c}}}{\mathcal {D}}^{\gamma }$ is Caputo fractional derivative, $0<\gamma \le 1$. Memory and heredity traits, which are complex behavioural patterns of biological systems, are goal of dealing with fractional order systems in our newly designed the Marburg virus transmission with public health education impact, these together allows us more realistic approach to biological systems. The memory function allows fractional order models to incorporate more knowledge from the past, allowing for more accurate prediction and translation. In addition, the hereditary property specifies the genetic profile, as well as the age and status of the immune system.

**Table 1:** Interpretation of parameters in the model.

Parameter	Interpretation	Value
$\Pi$	Recruitment rate of human	1–10
$\mu$	Natural humans mortality rate	$\frac{1}{87.7}$
$\nu$	Reduction of Marburg virus infection rate as a result of awareness	0.050
$\phi$	Public health education rate for Marburg virus infection	0.207
$\delta$	Marburg virus related death	0.001
$\varepsilon$	Fraction of Marburg exposed individuals becoming symptomatic infected	0.03
$\psi$	The rate of detecting unknown Marburg virus	0–1
*r*	Progression rate from exposed to infection	0.050
$\eta _{1}, \eta _{2}$	The rate at which undetected infected and Hospitalised individuals recover	0.1, 0.375
$\xi$	Rate at which the educated susceptible become susceptible	0.0021
$\vartheta$	The rate at which Marburg infected individuals Hospitalised	0–1
$\gamma _{1}$, $\gamma _{2}$	The modification parameters of ${I}_{u}$ and *H*, respectively	0.01, 0.002
$\beta$	The transmission rate	0–1

## Basic qualitative properties of the model

In this section, we give positivity, boundedness and existence of unique solution to projected model.

### Positivity and boundedness

#### Theorem 4.1

Assume $l_{i}\ge 0~(i=1,2,\cdot \cdot \cdot ,7)$, the solution of proposed system (2) is nonnegative and bounded.

#### Proof

Since the coefficients of (2) are positive constants, we have

$$\begin{aligned} \left\{ \begin{array}{llllllllll} {}^{{\mathfrak {c}}}{\mathcal {D}}^{\gamma }{\mathcal {S}}(t)\mid _{{\mathcal {S}}=0} =\Pi + \xi {\mathcal {P}}(t)\ge 0, \\ {}^{{\mathfrak {c}}}{\mathcal {D}}^{\gamma }{\mathcal {P}}(t)\mid _{{\mathcal {P}}=0} =\phi {\mathcal {S}}(t) \ge 0, \\ {}^{{\mathfrak {c}}}{\mathcal {D}}^{\gamma }{\mathcal {E}}(t)\mid _{{\mathcal {E}}=0} =\lambda {\mathcal {S}}(t) + \nu \lambda P(t) \ge 0,\\ {}^{{\mathfrak {c}}}{\mathcal {D}}^{\gamma }{\mathcal {I}}_{u}(t)\mid _{{\mathcal {I}}_{u}=0} =r(1-\varepsilon ){\mathcal {E}}(t)\ge 0,\\ {}^{{\mathfrak {c}}}{\mathcal {D}}^{\gamma }{\mathcal {I}}_{d}(t)\mid _{{\mathcal {I}}_{d}=0} =r\varepsilon {\mathcal {E}}(t)+\psi I_{u}(t)\ge 0, \\ {}^{{\mathfrak {c}}}{\mathcal {D}}^{\gamma }{\mathcal {H}}(t)\mid _{{\mathcal {H}}=0} =\vartheta {\mathcal {I}}_{d}(t)\ge 0, \\ {}^{{\mathfrak {c}}}{\mathcal {D}}^{\gamma }{\mathcal {R}} (t)\mid _{{\mathcal {R}} =0} =\eta _{1}{\mathcal {I}}_{u}+\eta _{2}{\mathcal {H}}(t)\ge 0,\\ \end{array} \right. \end{aligned}$$

Hence, the solution of (2) is non-negative. For the boundedness of solution, one get

$$\begin{aligned} {}^{{\mathfrak {c}}}{\mathcal {D}}^{\gamma }{\mathcal {N}}(t)=\Pi -\mu {\mathcal {N}}-\delta ({\mathcal {I}}_{u}+{\mathcal {I}}_{d}+{\mathcal {H}}), \end{aligned}$$

where

$$\begin{aligned} {\mathcal {N}}={\mathcal {S}}+{\mathcal {P}}+{\mathcal {E}}+{\mathcal {I}}_{u}+{\mathcal {I}}_{d}+{\mathcal {H}}+{\mathcal {R}}. \end{aligned}$$

Then, we can easily get

3 $$\begin{aligned} {}^{{\mathfrak {c}}}{\mathcal {D}}^{\gamma }{\mathcal {N}}(t)=\Pi -\mu {\mathcal {N}}. \end{aligned}$$

Apply the Laplace transform method to the inequality (3) with ${\mathcal {N}}(t_{0})\ge 0$, one can see that

$$\begin{aligned} {\mathcal {N}}\le \frac{\Pi }{s(s^{\gamma }+\mu )}+{\mathcal {N}}(0)\frac{s^{\gamma -1}}{s^{\gamma }+\mu }. \end{aligned}$$

Taking the Laplace inverse, we infer that

$$\begin{aligned} {\mathcal {N}}(t)\le {\mathcal {N}}(0)E_{\gamma ,1}(-\mu t^{\gamma })+ \Pi t^{\gamma }E_{\gamma ,\gamma +1}(-\mu t^{\gamma }), \end{aligned}$$

from $E_{\gamma ,1}(-\mu t^{\gamma }), E_{\gamma ,\gamma +1}(-\mu t^{\gamma })$ are the series of the Mittag-Leffler function which converges for any argument. Hence, the solution to the model is bounded.

### Basic reproduction number, disease-free equilibrium

Let some parameters of system (2) be

$$\begin{aligned} r_{1}=\lambda +\mu +\phi ,~~r_{2}=\nu \lambda +\mu +\xi ,~~ r_{3}=r + \mu , ~~r_{4}=(\eta _{1}+\psi +\mu +\delta ),~~r_{5}=\vartheta +\mu +\delta ,~~r_{6}=\eta _{2}+\delta + \mu , \end{aligned}$$

and the right side of the equation be zero, that is

4 $$\begin{aligned} \left\{ \begin{array}{llllllllll} \Pi + \xi {\mathcal {P}}(t)-r_{1}{\mathcal {S}}(t)=0, \\ \phi {\mathcal {S}}(t)-r_{2} {\mathcal {P}}(t)=0, \\ \lambda {\mathcal {S}}(t) + \nu \lambda P(t)-r_{3} {\mathcal {E}}(t)=0, \\ r(1-\varepsilon ){\mathcal {E}}(t)-r_{4}{\mathcal {I}}_{u}(t)=0,\\ r\varepsilon {\mathcal {E}}(t)+\psi I_{u}(t)-r_{5}{\mathcal {I}}_{d}(t)=0, \\ \vartheta {\mathcal {I}}_{d}(t)- r_{6} {\mathcal {H}}(t)=0, \\ \eta _{1}{\mathcal {I}}_{u}+\eta _{2}{\mathcal {H}}(t)-\mu {\mathcal {R}}(t)=0. \end{array} \right. \end{aligned}$$

Then disease-free equilibrium of model (2) is given by

$$\begin{aligned} E_{0}=\left( \frac{\Pi }{\mu },0,0,0,0,0,0\right) , \end{aligned}$$

and the endemic disease equilibrium is given by

$$\begin{aligned} ({\mathcal {S}}^{*}, {\mathcal {P}}^{*}, {\mathcal {E}}^{*}, {\mathcal {I}}_{u}^{*}, {\mathcal {I}}_{d}^{*}, {\mathcal {H}}^{*}, {\mathcal {R}}^{*}), \end{aligned}$$

where

$$\begin{aligned}{} & {} {\mathcal {S}}^{*}=\frac{\Pi r_{2}}{r_{1}r_{2}-\xi \phi },~~ {\mathcal {P}}^{*}=\frac{ \phi {\mathcal {S}}^{*}}{ r_{2}}, ~~{\mathcal {E}}^{*}=\frac{\lambda (r_{2}+\phi \nu ) {\mathcal {S}}^{*}}{ r_{2}r_{3}},~~ {\mathcal {I}}_{u}^{*}=\frac{\lambda r(r_{2}+\phi \nu )(1-\varepsilon ) {\mathcal {S}}^{*}}{ r_{2}r_{3}r_{4}},\\{} & {} {\mathcal {I}}_{d}^{*}=\frac{\lambda r(r_{2}+\phi \nu )[\phi (1-\varepsilon )+\varepsilon r_{4}] {\mathcal {S}}^{*}}{ r_{2}r_{3}r_{4}},~~ {\mathcal {H}}^{*}=\frac{\lambda r\vartheta (r_{2}+\phi \nu )[\phi (1-\varepsilon )+\varepsilon r_{4}] {\mathcal {S}}^{*}}{ r_{2}r_{3}r_{4}r_{5}r_{6}}, \\{} & {} {\mathcal {R}}^{*}=\frac{\lambda r (r_{2}+\phi \nu )[(\psi \eta _{2}\vartheta +\eta _{1}r_{5}r_{6})(1-\varepsilon )+\varepsilon \eta _{2}r_{4}\vartheta ] {\mathcal {S}}^{*}}{ r_{2}r_{3}r_{4}r_{5}r_{6}}. \end{aligned}$$

Let $K= ({\mathcal {S}},{\mathcal {P}},{\mathcal {E}}, {\mathcal {I}}_{u}, {\mathcal {I}}_{d}, {\mathcal {H}}, {\mathcal {R}})^{T }$, it gives

$$\begin{aligned} \frac{dK}{dt}=F-V, \end{aligned}$$

where

$$\begin{aligned}{} & {} F(x)=\left[ \begin{matrix} 0 &{} 0 &{} 0 &{} 0 &{} 0 &{} 0 &{} 0 \\ 0 &{} 0 &{} 0 &{} 0 &{} 0 &{} 0 &{} 0 \\ 0 &{} 0 &{} 0 &{} \beta \gamma _{1} &{} \beta \gamma _{2} &{} \beta &{} 0 \\ 0 &{} 0 &{} 0 &{} 0 &{} 0 &{} 0 &{} 0 \\ 0 &{} 0 &{} 0 &{} 0 &{} 0 &{} 0 &{} 0 \\ 0 &{} 0 &{} 0 &{} 0 &{} 0 &{} 0 &{} 0 \\ 0 &{} 0 &{} 0 &{} 0 &{} 0 &{} 0 &{} 0 \end{matrix} \right] ,\\{} & {} V(x)=\left[ \begin{matrix} \mu +\phi &{} -\xi &{} 0 &{} 0 &{} 0 &{} 0 &{} 0 \\ -\phi &{} \mu +\xi &{} 0 &{} 0 &{} 0 &{} 0 &{} 0 \\ 0 &{} 0 &{}\mu + r &{} 0 &{} 0 &{} 0 &{} 0\\ 0 &{} 0 &{} -r(1-\varepsilon ) &{} \eta _{1}+\psi +\mu +\delta &{} 0 &{} 0 &{} 0 \\ 0 &{} 0 &{} -r\varepsilon &{} -\psi &{} \vartheta +\mu +\delta &{} 0 &{} 0\\ 0 &{} 0 &{} 0 &{} 0 &{} -\vartheta &{}\eta _{2}+\mu +\delta &{} 0\\ 0 &{} 0 &{} 0 &{} -\eta _{1} &{} 0 &{} -\eta _{2} &{} \mu \end{matrix} \right] . \end{aligned}$$

From $R_{0}= \rho (FV^{-1})$ ($\rho (\cdot )$ represents the spectral radius), one observes

$$\begin{aligned} \begin{aligned} R_{0}=&\frac{\beta \gamma _{1}r(1-\varepsilon ) }{(r+\mu )(\delta +\eta _{1}+\psi +\mu )}+\frac{\beta \gamma _{2}r[\psi +(\delta +\eta _{1}+\mu )\varepsilon ] }{(r+\mu )(\delta +\eta _{1}+\psi +\mu )(\delta + \vartheta +\mu )}\\&+\frac{\beta \vartheta r[\psi +(\delta +\eta _{1}+\mu )\varepsilon ] }{(r+\mu )(\delta +\eta _{1}+\psi +\mu )(\delta + \vartheta +\mu ) (\delta + \eta _{2}+\mu ) }. \end{aligned} \end{aligned}$$

#### Theorem 4.2

The disease-free equilibrium $E_{0}$ of (2) is globally asymptotically stable if $R_{0}<1$ and is unstable if $R_{0}>1$.

#### Proof

Consider a Lyapunov function:

$$\begin{aligned} \begin{aligned} {\textbf{L}}(t)=&{\mathcal {P}}(t) +l_{1}{\mathcal {E}}(t) + l_{2}{\mathcal {I}}_{u}(t)+ l_{3}{\mathcal {I}}_{d}(t) +l_{4}{\mathcal {H}}(t)-\phi {\mathcal {S}}(t), \end{aligned} \end{aligned}$$

where $a_{i}~(i = 1, 2, \cdot \cdot \cdot , 4)$ are positive constants. Thus, one gets

$$\begin{aligned} \begin{aligned} {}^{{\mathfrak {c}}}{\mathcal {D}}^{\gamma }{\textbf{L}}(t) =&(l_{1}\nu \lambda -r_{2}){\mathcal {P}}(t)+(l_{2}r(1-\varepsilon )+l_{3}r\varepsilon -l_{1}r_{3}){\mathcal {E}}(t)\\&+(l_{3}\psi +\beta \gamma _{1}l_{1}-l_{2}r_{4}){\mathcal {I}}_{u}(t)(t) +(l_{4}\vartheta +\beta \gamma _{2}l_{1}-l_{3}r_{5}){\mathcal {I}}_{d}(t)\\&+( \beta \gamma _{3}l_{1}-l_{4}r_{6}){\mathcal {H}}(t). \end{aligned} \end{aligned}$$

If

$$\begin{aligned}{} & {} l_{1}=\frac{r_{2} }{ \nu (\beta \gamma _{1}+\beta \gamma _{2}+\beta )}, ~~l_{2}=\frac{r_{2}r_{3}}{\nu (\beta \gamma _{1}+\beta \gamma _{2}+\beta )r(1-\varepsilon ) }-\frac{l_{3} \varepsilon }{(1-\varepsilon )},~~ \\{} & {} l_{3}=\frac{r_{2}r_{3} r_{4}-\beta \gamma _{1}r_{2}r(1-\varepsilon )}{\nu (\beta \gamma _{1}+\beta \gamma _{2}+\beta )r(1-\varepsilon ) [rr_{4}\varepsilon +\psi r(1-\varepsilon )]},~~l_{4}=\frac{\beta l_{1}}{ r_{6}}, \end{aligned}$$

then we get

$$\begin{aligned} \begin{aligned} {}^{{\mathfrak {c}}}{\mathcal {D}}^{\gamma }{\textbf{L}}(t)\le&\Big \{ \frac{\beta \gamma _{1}r_{2}r_{5}r(1-\varepsilon )-r_{2}r_{3}r_{4} r_{5}}{\nu (\beta \gamma _{1}+\beta \gamma _{2}+\beta )[rr_{4}\varepsilon +\psi r(1-\varepsilon )]}\\&+\frac{\beta \gamma _{3}r_{2}\vartheta +\beta \gamma _{2}r_{2}r_{6}}{r_{6}\nu (\beta \gamma _{1}+\beta \gamma _{2}+\beta }\Big \} I_{d}(t)\\ \le&\Big \{ \frac{(r+\mu )(\delta +\eta _{1}+\psi +\mu )(\delta + \vartheta +\mu ) (\delta + \eta _{2}+\mu ) }{\nu (\beta \gamma _{1}+\beta \gamma _{2}+\beta )r[\psi +(\eta _{1}+\mu +\delta )\varepsilon ](\eta _{2}+\mu +\delta )}\Big \} (R_{0}-1)I_{d}(t). \end{aligned} \end{aligned}$$

Hence, if $R_{0}<1$, one gets ${}^{{\mathfrak {c}}}{\mathcal {D}}^{\gamma }{\textbf{L}}\le 0.$ Using the LaSalle’s invariant principle, $E_{0}$ is globally asymptotically stable if $R_{0}<1$ and is unstable otherwise.

## Existence and uniqueness

Let us write the system (2) in the compact form for easy description as follows:

5 $$\begin{aligned} \left\{ \begin{array}{llllllllll} {}^{{\mathfrak {c}}}{\mathcal {D}}^{\gamma }{\mathcal {S}}(t) ={\mathfrak {G}}_{1}(t, {\mathcal {S}},{\mathcal {P}},{\mathcal {E}},{\mathcal {I}}_{u},{\mathcal {I}}_{d},{\mathcal {H}},{\mathcal {R}} ), \\ {}^{{\mathfrak {c}}}{\mathcal {D}}^{\gamma }{\mathcal {P}}(t) ={\mathfrak {G}}_{2}(t, {\mathcal {S}},{\mathcal {P}},{\mathcal {E}},{\mathcal {I}}_{u},{\mathcal {I}}_{d},{\mathcal {H}},{\mathcal {R}} ), \\ {}^{{\mathfrak {c}}}{\mathcal {D}}^{\gamma }{\mathcal {E}}(t) ={\mathfrak {G}}_{3}(t, {\mathcal {S}},{\mathcal {P}},{\mathcal {E}},{\mathcal {I}}_{u},{\mathcal {I}}_{d},{\mathcal {H}},{\mathcal {R}} ), \\ {}^{{\mathfrak {c}}}{\mathcal {D}}^{\gamma }{\mathcal {I}}_{u}(t) ={\mathfrak {G}}_{4}(t, {\mathcal {S}},{\mathcal {P}},{\mathcal {E}},{\mathcal {I}}_{u},{\mathcal {I}}_{d},{\mathcal {H}},{\mathcal {R}} ),\\ {}^{{\mathfrak {c}}}{\mathcal {D}}^{\gamma }{\mathcal {I}}_{d}(t) ={\mathfrak {G}}_{5}(t, {\mathcal {S}},{\mathcal {P}},{\mathcal {E}},{\mathcal {I}}_{u},{\mathcal {I}}_{d},{\mathcal {H}},{\mathcal {R}} ),\\ {}^{{\mathfrak {c}}}{\mathcal {D}}^{\gamma }{\mathcal {H}}(t) ={\mathfrak {G}}_{6}(t, {\mathcal {S}},{\mathcal {P}},{\mathcal {E}},{\mathcal {I}}_{u},{\mathcal {I}}_{d},{\mathcal {H}},{\mathcal {R}} ),\\ {}^{{\mathfrak {c}}}{\mathcal {D}}^{\gamma }{\mathcal {R}} (t) ={\mathfrak {G}}_{7}(t, {\mathcal {S}},{\mathcal {P}},{\mathcal {E}},{\mathcal {I}}_{u},{\mathcal {I}}_{d},{\mathcal {H}},{\mathcal {R}} ),\\ {\mathcal {S}}(0)=l_{1},{\mathcal {P}}(0)=l_{2},{\mathcal {E}}(0)=l_{3},{\mathcal {I}}_{u}(0)=l_{4},\\ {\mathcal {I}}_{d}(0)=l_{5},{\mathcal {H}}(0)=l_{6},{\mathcal {R}}(0)=l_{7}, \end{array} \right. \end{aligned}$$

Let ${\mathfrak {C}}[0,T]$ be the set of all real continuous function on the interval [0, *T*] with the norm $||z||=\sup \{|z|,z\in {\mathfrak {C}}[0,T]\}.$ Clearly, ${\mathfrak {C}}[0,T]$ is a Banach space. Define ${\mathfrak {P}}=\{z\in {\mathfrak {C}}:z(x)\ge 0, \forall x\in [0,T]\}.$

We show the analysis for ${\mathcal {S}}$ and for others it is similar. Hence we consider the initial value system

6 $$\begin{aligned} \left\{ \begin{array}{llllllllll} {}^{{\mathfrak {c}}}{\mathcal {D}}^{\gamma }{\mathcal {S}}(t) ={\mathfrak {G}}_{1}(t, {\mathcal {S}},{\mathcal {P}},{\mathcal {E}},{\mathcal {I}}_{u},{\mathcal {I}}_{d},{\mathcal {H}},{\mathcal {R}} ), \\ {\mathcal {S}}(0)=l_{1}, \end{array} \right. \end{aligned}$$

according to the definition and lemma of Caputo fractional calculus, we can obtain the equivalent integral solution of the above system (6) as

7 $$\begin{aligned} {\mathcal {S}}(x)=\int ^{x}_{0}\frac{(x-y)^{\gamma -1}}{\Gamma (\gamma )} {\mathfrak {G}}_{1}(y, {\mathcal {S}}(y),{\mathcal {P}}(y),{\mathcal {E}}(y),{\mathcal {I}}_{u}(y),{\mathcal {I}}_{d}(y),{\mathcal {H}}(y),{\mathcal {R}} (y))\textrm{d}y+l_{1}. \end{aligned}$$

### Theorem 5.1

Let $\frac{T^{\gamma }{\mathfrak {L}}}{\Gamma (\gamma +1)}<1.$ Assume that

$({\textbf{H}}_{1})$ ${\mathfrak {G}}_{1}(y, {\mathcal {S}},{\mathcal {P}},{\mathcal {E}},{\mathcal {I}}_{u},{\mathcal {I}}_{d},{\mathcal {H}},{\mathcal {R}})$ is continuous function;

$({\textbf{H}}_{2})$ for any ${\mathcal {S}}_{1}, {\mathcal {S}}_{2}\in C[0,T],$ there exists a constant ${\mathfrak {L}}>0$, such that

$$\begin{aligned} |{\mathfrak {G}}_{1}(x, {\mathcal {S}}_{1},{\mathcal {P}},{\mathcal {E}},{\mathcal {I}}_{u},{\mathcal {I}}_{d},{\mathcal {H}},{\mathcal {R}} )-{\mathfrak {G}}_{1}(x, {\mathcal {S}}_{2},{\mathcal {P}},{\mathcal {E}},{\mathcal {I}}_{u},{\mathcal {I}}_{d},{\mathcal {H}},{\mathcal {R}})|\le {\mathfrak {L}} | {\mathcal {S}}_{1}-{\mathcal {S}}_{2}|. \end{aligned}$$

Then the system (6) has a unique solution.

### Proof

Define an operator ${\textbf{T}}:{\mathfrak {B}}\rightarrow {\mathfrak {C}}$ by

$$\begin{aligned} {\textbf{T}}{\mathcal {S}}(x)=\int ^{x}_{0}\frac{(x-y)^{\gamma -1}}{\Gamma (\gamma )} {\mathfrak {G}}_{1}(y, {\mathcal {S}}(y),{\mathcal {P}}(y),{\mathcal {E}}(y),{\mathcal {I}}_{u}(y),{\mathcal {I}}_{d}(y),{\mathcal {H}}(y),{\mathcal {R}} (y))\textrm{d}y+l_{1}, \end{aligned}$$

from (7), ${\mathcal {S}}$ is the unique solution for system (6) if and only if ${\mathcal {S}}$ is the unique solution for ${\textbf{T}}{\mathcal {S}}={\mathcal {S}}.$

Step 1: Prove that the operator ${\textbf{T}}$ is completely continuous by Arzela-Ascoli theorem.

(1) Prove that ${\textbf{T}} \mathbf {\Omega }$ is bounded, where $\mathbf {\Omega }$ is a bounded subset of ${\mathfrak {B}}$. For any ${\mathcal {S}},{\mathcal {P}},{\mathcal {E}},{\mathcal {I}}_{u},{\mathcal {I}}_{d},{\mathcal {H}},{\mathcal {R}}\in \mathbf {\Omega },$ by the condition $({\textbf{H}}_{1})$, $\exists {\mathfrak {m}}>0,$ such that

$$\begin{aligned} 0\le {\mathfrak {G}}_{1}(y, {\mathcal {S}}(y),{\mathcal {P}}(y),{\mathcal {E}}(y),{\mathcal {I}}_{u}(y),{\mathcal {I}}_{d}(y),{\mathcal {H}}(y),{\mathcal {R}} (y))\le {\mathfrak {m}}. \end{aligned}$$

Then one can see that

$$\begin{aligned} |{\textbf{T}} {\mathcal {S}}(x)|= \Big |\int ^{x}_{0}\frac{(x-y)^{\gamma -1}}{\Gamma (\gamma )} {\mathfrak {G}}_{1}(y, {\mathcal {S}}(y),{\mathcal {P}}(y),{\mathcal {E}}(y),{\mathcal {I}}_{u}(y),{\mathcal {I}}_{d}(y),{\mathcal {H}}(y),{\mathcal {R}} (y))\textrm{d}y+ l_{1}\Big |\le \frac{{\mathfrak {m}}T^{\gamma }}{\Gamma (\gamma +1)}+ l_{1}. \end{aligned}$$

(2) Show that ${\textbf{T}} \mathbf {\Omega }$ is equicontinuous.

Duo to $\frac{(x-y)^{\gamma -1}}{\Gamma (\gamma )}$ is uniformly continuous on any bounded subset $\mathbf {\Omega }$, for any $\varrho >0, \exists \varsigma<(\frac{\Gamma (\gamma +1)\mathbf {\varrho }}{2{\mathfrak {m}}})^{\frac{1}{\gamma }}, x_{1},x_{2}\in [0,T],|x_{2}-x_{1}|< \varsigma ,$ such that

$$\begin{aligned} |\frac{(x_{2}-y)^{\gamma -1}}{\Gamma (\gamma )}-\frac{(x_{1}-y)^{\gamma -1}}{\Gamma (\gamma )}| <\frac{\varrho }{2T{\mathfrak {m}}}. \end{aligned}$$

Then it holds that

$$\begin{aligned} \begin{aligned} |{\textbf{T}}{\mathcal {S}}(x_{2})-{\textbf{T}}{\mathcal {S}}(x_{1})| \le&\int ^{x_{1}}_{0}|\frac{(x_{2}-y)^{\gamma -1}}{\Gamma (\gamma )}-\frac{(x_{1}-y)^{\gamma -1}}{\Gamma (\gamma )}|\\&\times {\mathfrak {G}}_{1}(y, {\mathcal {S}}(y),{\mathcal {P}}(y),{\mathcal {E}}(y),{\mathcal {I}}_{u}(y),{\mathcal {I}}_{d}(y),{\mathcal {H}}(y),{\mathcal {R}} (y))\textrm{d}y\\&+ \int ^{x_{2}}_{x_{1}}\frac{(x_{2}-y)^{\gamma -1}}{\Gamma (\gamma )}{\mathfrak {G}}_{1}(y, {\mathcal {S}}(y),{\mathcal {P}}(y),{\mathcal {E}}(y),{\mathcal {I}}_{u}(y),{\mathcal {I}}_{d}(y),{\mathcal {H}}(y),{\mathcal {R}} (y))\textrm{d}y \\ \le&\frac{\varrho }{2T{\mathfrak {m}}}T{\mathfrak {m}}+ \frac{\varsigma ^{\gamma }{\mathfrak {m}}}{\Gamma (\gamma +1)} = \varepsilon . \end{aligned} \end{aligned}$$

(3) State ${\textbf{T}}:{\mathfrak {B}}\rightarrow {\mathfrak {B}}$ is continuous.

From $({\textbf{H}}_{1})$ and the function $\frac{(x-y)^{\gamma -1}}{\Gamma (\gamma )}>0$, we get ${\textbf{T}}: {\mathfrak {B}}\rightarrow {\mathfrak {B}}$. Set ${\mathcal {S}}_{n},{\mathcal {S}} \in {\mathfrak {B}}$ and ${\mathcal {S}}_{n}\rightarrow {\mathcal {S}}$ as $n \rightarrow +\infty$. From the Lebesgue dominated convergence theorem and the continuity of the function ${\mathfrak {G}}_{1}$, we can get

$$\begin{aligned} \begin{aligned}{}&\lim _{n\rightarrow +\infty } {\mathfrak {G}}_{1}(y, {\mathcal {S}}_{n}(y),{\mathcal {P}}(y),{\mathcal {E}}(y),{\mathcal {I}}_{u}(y),{\mathcal {I}}_{d}(y),{\mathcal {H}}(y),{\mathcal {R}} (y))\\&={\mathfrak {G}}_{1}(y, {\mathcal {S}}(y),{\mathcal {P}}(y),{\mathcal {E}}(y),{\mathcal {I}}_{u}(y),{\mathcal {I}}_{d}(y),{\mathcal {H}}(y),{\mathcal {R}} (y)). \end{aligned} \end{aligned}$$

Then we get $\lim _{n\rightarrow +\infty }{\textbf{T}}{\mathcal {S}}_{n}(y)\rightarrow {\textbf{T}}{\mathcal {S}}(y).$

Step 2: Show that the system (6) has a unique solution.

For any ${\mathcal {S}}_{1}, {\mathcal {S}}_{2}\in {\mathcal {C}},$ by $({\textbf{H}}_{2})$, we observe that

$$\begin{aligned} \begin{aligned} |{\textbf{T}}{\mathcal {S}}_{1}(x)-{\textbf{T}}{\mathcal {S}}_{2}(x)| \le&\int ^{x}_{0}\frac{(x-y)^{\gamma -1}}{\Gamma (\gamma )} |{\mathfrak {G}}_{1}(y, {\mathcal {S}}_{1}(y),{\mathcal {P}}(y),{\mathcal {E}}(y),{\mathcal {I}}_{u}(y),{\mathcal {I}}_{d}(y),{\mathcal {H}}(y),{\mathcal {R}} (y))\\&-{\mathfrak {G}}_{1}(y, {\mathcal {S}}_{2}(y),{\mathcal {P}}(y),{\mathcal {E}}(y),{\mathcal {I}}_{u}(y),{\mathcal {I}}_{d}(y),{\mathcal {H}}(y),{\mathcal {R}} (y))|\textrm{d}y \le \frac{T^{\gamma }}{\Gamma (\gamma +1)}{\mathfrak {L}} | {\mathcal {S}}_{1}-{\mathcal {S}}_{2} |. \end{aligned} \end{aligned}$$

Then by $\frac{T^{\gamma }{\mathfrak {L}}}{\Gamma (\gamma +1)}<1,$ it gives

$$\begin{aligned} \Vert {\textbf{T}}{\mathcal {S}}_{1}-{\textbf{T}}{\mathcal {S}}_{2}\Vert \le \Vert {\mathcal {S}}_{1}-{\mathcal {S}}_{2}\Vert . \end{aligned}$$

From contraction mapping principle, ${\textbf{T}}$ has a unique fixed point. That is, (6) has a unique solution.

## HU stability

### Definition 6.1

(HU stability) $({\textbf{H}}_{3})$ If $\exists {\mathfrak {K}}$, satisfying for any $d >0$,

8 $$\begin{aligned} |^{{\mathfrak {c}}}{\mathcal {D}}^{\gamma }{\mathcal {S}}(t)-{\mathfrak {G}}_{1}(y, {\mathcal {S}}(y),{\mathcal {P}}(y),{\mathcal {E}}(y),{\mathcal {I}}_{u}(y),{\mathcal {I}}_{d}(y),{\mathcal {H}}(y),{\mathcal {R}} (y))|\le d, \end{aligned}$$

and for the unique solution $\widetilde{{\mathcal {S}}}$ of model (6) such that

$$\begin{aligned} \Vert {\mathcal {S}}-\widetilde{{\mathcal {S}}} \Vert \le {\mathfrak {K}} d, \end{aligned}$$

then, (3.4) is HU stable.

### Theorem 6.1

Assume that $\frac{T^{\gamma }{\mathfrak {L}}}{\Gamma (\gamma +1)}<1,$ and $({\textbf{H}}_{2})$ hold and

$({\textbf{H}}_{4})$ there exist a function ${\mathfrak {h}}_{1}$ such that

$$\begin{aligned} |{\mathfrak {h}}_{1}(t)|\le d, ~~^{{\mathfrak {c}}}{\mathcal {D}}^{\gamma }{\mathcal {S}}(t)={\mathfrak {G}}_{1}(y, {\mathcal {S}}_{1}(y),{\mathcal {P}}(y),{\mathcal {E}}(y),{\mathcal {I}}_{u}(y),{\mathcal {I}}_{d}(y),{\mathcal {H}}(y),{\mathcal {R}} (y))+{\mathfrak {h}}_{1}(t). \end{aligned}$$

Then, (6) is HU stable.

### Proof

For solution ${\mathcal {S}}$ of system (6), according to $({\textbf{H}}_{4})$, it gives

$$\begin{aligned} {\mathcal {S}}(x)=l_{0}+\int ^{x}_{0}\frac{(x-y)^{\gamma -1}}{\Gamma (\gamma )} {\mathfrak {G}}_{1}(y, {\mathcal {S}}(y),{\mathcal {P}}(y),{\mathcal {E}}(y),{\mathcal {I}}_{u}(y),{\mathcal {I}}_{d}(y),{\mathcal {H}}(y),{\mathcal {R}} (y))+\int ^{x}_{0}\frac{(x-y)^{\gamma -1}}{\Gamma (\gamma )} {\mathfrak {h}}_{1}(y)\textrm{d}y, \end{aligned}$$

Hence, one gets

$$\begin{aligned} \begin{aligned} |{\mathcal {S}}(x)-l_{1}-\int ^{x}_{0}\frac{(x-y)^{\gamma -1}}{\Gamma (\gamma )}&{\mathfrak {G}}_{1}(y, {\mathcal {S}}(y),{\mathcal {P}}(y),{\mathcal {E}}(y), {\mathcal {I}}_{u}(y),{\mathcal {I}}_{d}(y),{\mathcal {H}}(y),{\mathcal {R}} (y))\textrm{d}y|\\&\le \int ^{x}_{0}\frac{(x-y)^{\gamma -1}}{\Gamma (\gamma )} {\mathfrak {h}}_{1}(y)\textrm{d}y=\frac{T^{\gamma } }{\Gamma (\gamma +1)} d. \end{aligned} \end{aligned}$$

Let $\widetilde{{\mathcal {S}}}$ be unique solution to system (6), we have

$$\begin{aligned} \begin{aligned} \Big |{\mathcal {S}}(x)-\widetilde{{\mathcal {S}}}(x)\Big |=&\Big |{\mathcal {S}}(x)-l_{1} -\int ^{x}_{0}\frac{(x-y)^{\gamma -1}}{\Gamma (\gamma )} {\mathfrak {G}}_{1}(y, \widetilde{{\mathcal {S}}}(y), {\mathcal {P}}(y), {\mathcal {E}}(y), {\mathcal {I}}_{u}(y), {\mathcal {I}}_{d}(y), {\mathcal {H}}(y), {\mathcal {R}}(y))\textrm{d}y\Big |\\ \le&\Big |{\mathcal {S}}(x)- l_{1} -\int ^{x}_{0}\frac{(x-y)^{\gamma -1}}{\Gamma (\gamma )} {\mathfrak {G}}_{1}(y, {\mathcal {S}}(y),{\mathcal {P}}(y),{\mathcal {E}}(y),{\mathcal {I}}_{u}(y),{\mathcal {I}}_{d}(y),{\mathcal {H}}(y),{\mathcal {R}} (y))\textrm{d}y\Big |\\&+\int ^{x}_{0}\frac{(x-y)^{\gamma -1}}{\Gamma (\gamma )} \Big |{\mathfrak {G}}_{1}(y, {\mathcal {S}}(y),{\mathcal {P}}(y),{\mathcal {E}}(y),{\mathcal {I}}_{u}(y),{\mathcal {I}}_{d}(y),{\mathcal {H}}(y),{\mathcal {R}} (y))\\&-{\mathfrak {G}}_{1}(y, \widetilde{{\mathcal {S}}}(y), {\mathcal {P}}(y), {\mathcal {E}}(y), {\mathcal {I}}_{u}(y), {\mathcal {I}}_{d}(y),{\mathcal {H}}(y),{\mathcal {R}}(y))\Big |\textrm{d}y\\ \le&\frac{T^{\gamma }}{\Gamma (\gamma +1)} d +\frac{T^{\gamma }{\mathfrak {L}}}{\Gamma (\gamma +1)} |{\mathcal {S}}-\widetilde{{\mathcal {S}}}|. \end{aligned} \end{aligned}$$

Hence, we deduce

$$\begin{aligned} \Vert {\mathcal {S}}(t)-\widetilde{{\mathcal {S}}}(t)\Vert \le {\mathfrak {K}} d, \end{aligned}$$

where ${\mathfrak {K}}=\frac{T^{\gamma }}{ \Gamma (\gamma +1)-T^{\gamma }{\mathfrak {L}}}.$ By $({\textbf{H}}_{3})$ of Definition 6.1, the system (6) is HU stable.

## Numerical simulation

In this section, the numerical solution of system (2) is given by corrector-predictor iterative scheme in Caputos sense. Let

$$\begin{aligned} \left\{ \begin{array}{llllllllll} {\mathcal {U}}=(S, P, E,I_{u},I_{d},H,R)^{T},\\ {\mathcal {U}}(0)={\mathcal {U}}_{0}=({\mathcal {U}}_{1},{\mathcal {U}}_{2}, {\mathcal {U}}_{3},{\mathcal {U}}_{4},{\mathcal {U}}_{5},{\mathcal {U}}_{6},{\mathcal {U}}_{7})\\ ~~~~~~=(S_{0}, P_{0}, E_{0},I_{u0},I_{d0},H_{0},R_{0})^{T},\\ {\mathfrak {L}}_{1}(t,{\mathcal {U}})=\Pi + \xi {\mathcal {P}}(t)-(\lambda +\mu +\phi ){\mathcal {S}}(t), \\ {\mathfrak {L}}_{2}(t,{\mathcal {U}})=\phi {\mathcal {S}}(t)-(\nu \lambda +\mu +\xi ){\mathcal {P}}(t),\\ {\mathfrak {L}}_{3}(t,{\mathcal {U}})=\lambda {\mathcal {S}}(t) + \nu \lambda P(t)-(r + \mu ) {\mathcal {E}}(t), \\ {\mathfrak {L}}_{4}(t,{\mathcal {U}})=r(1-\varepsilon ){\mathcal {E}}(t)-(\eta _{1}+\psi +\mu +\delta ){\mathcal {I}}_{u}(t), \\ {\mathfrak {L}}_{5}(t,{\mathcal {U}})=r\varepsilon {\mathcal {E}}(t)+\psi I_{u}(t)-(\vartheta +\mu +\delta ){\mathcal {I}}_{d}(t), \\ {\mathfrak {L}}_{6}(t,{\mathcal {U}}) =\vartheta {\mathcal {I}}_{d}(t)- (\eta _{2}+\delta + \mu ){\mathcal {H}}(t), \\ {\mathfrak {L}}_{7}(t,{\mathcal {U}})= \eta _{1}{\mathcal {I}}_{u}+\eta _{2}{\mathcal {H}}(t)-\mu {\mathcal {R}}(t),\\ {\mathfrak {L}}(t,{\mathcal {U}})=({\mathfrak {L}}_{1}(t,{\mathcal {U}}),{\mathfrak {L}}_{2}(t,{\mathcal {U}}), \cdot \cdot \cdot ,{\mathfrak {L}}_{7}(t,{\mathcal {U}}))^{T}, \end{array} \right. \end{aligned}$$

the proposed model can be written into the following one as

9 $$\begin{aligned} \left\{ \begin{array}{llllllllll} {}^{{\mathfrak {c}}}{\mathcal {D}}^{\gamma } {\mathcal {U}}={\mathfrak {L}}(t,{\mathcal {U}}),\\ {\mathcal {U}}(0)={\mathcal {U}}_{0}. \end{array} \right. \end{aligned}$$

Choose step length ${\textbf{h}}=\frac{T}{M}$ and using the integral equation equivalent to system (2), ${\mathcal {U}}_{a}(t_{j+1})~( j=0,1,\cdot \cdot \cdot ,n)$ can be calculated

$$\begin{aligned} \begin{aligned} {\mathcal {U}}_{a}(t_{j+1})=&\frac{{\textbf{h}}^{\gamma }}{\Gamma (\gamma +2)}\Big [\sum ^{n}_{j=0} d_{j,n+1} {\mathfrak {L}}(t_{j}, {\mathcal {U}}_{a}(t_{j}))+ {\mathfrak {L}}(t_{n+1}, {\mathcal {U}}^{p}_{a}(t_{n+1}))\Big ]+ {\mathcal {U}}_{0}, \end{aligned} \end{aligned}$$

where

$$\begin{aligned} d_{j,n+1}= {\left\{ \begin{array}{ll} \begin{array}{l} n^{\gamma +1}-(n-\gamma )(n+1)^{\gamma },~~~ j=0,\\ (n-j+2)^{\gamma +1}+(n-j)^{\gamma +1}-2(n-j+1)^{\gamma +1}, ~~~1\le j\le n,\\ 1,~~~j=n+1, \end{array} \end{array}\right. } \end{aligned}$$

The Predictor formula is derived as follows:

$$\begin{aligned} \begin{aligned} {\mathcal {U}}^{p}_{a}(t_{n+1})= \frac{1}{\Gamma (\gamma +1)}\sum ^{n}_{j=0} {\mathfrak {h}}^{\gamma } [(n-j+1)^{\gamma }-(n-j)^{\gamma }]{\mathfrak {L}}(t_{j}, {\mathcal {U}}_{a}(t_{j}))+ {\mathcal {U}}_{0}, \end{aligned} \end{aligned}$$

Thus the corrector formula for system (2) is

$$\begin{aligned} \begin{aligned}{}&{}^{{\mathfrak {c}}}{\mathcal {D}}^{\gamma }{\mathcal {S}}(t_{j+1})= \frac{{\textbf{h}}^{\gamma }}{\Gamma (\gamma +2)}\Big [\sum ^{n}_{j=0} d_{j,n+1} {\mathfrak {L}}_{1}(t_{j}, {\mathcal {U}}_{a}(t_{j}))+ {\mathfrak {L}}_{1}(t_{n+1}, {\mathcal {U}}^{p}_{a}(t_{n+1}))\Big ]+ l_{1},\\&{}^{{\mathfrak {c}}}{\mathcal {D}}^{\gamma }{\mathcal {P}}(t_{j+1})= \frac{{\textbf{h}}^{\gamma }}{\Gamma (\gamma +2)}\Big [\sum ^{n}_{j=0} d_{j,n+1} {\mathfrak {L}}_{2}(t_{j}, {\mathcal {U}}_{a}(t_{j}))+ {\mathfrak {L}}_{2}(t_{n+1}, {\mathcal {U}}^{p}_{a}(t_{n+1}))\Big ]+ l_{2},\\&{}^{{\mathfrak {c}}}{\mathcal {D}}^{\gamma }{\mathcal {E}}(t_{j+1})= \frac{{\textbf{h}}^{\gamma }}{\Gamma (\gamma +2)}\Big [\sum ^{n}_{j=0} d_{j,n+1} {\mathfrak {L}}_{3}(t_{j}, {\mathcal {U}}_{a}(t_{j}))+ {\mathfrak {L}}_{3}(t_{n+1}, {\mathcal {U}}^{p}_{a}(t_{n+1}))\Big ]+ l_{3},\\&{}^{{\mathfrak {c}}}{\mathcal {D}}^{\gamma }{\mathcal {I}}_{u}(t_{j+1})= \frac{{\textbf{h}}^{\gamma }}{\Gamma (\gamma +2)}\Big [\sum ^{n}_{j=0} d_{j,n+1} {\mathfrak {L}}_{4}(t_{j}, {\mathcal {U}}_{a}(t_{j}))+ {\mathfrak {L}}_{4}(t_{n+1}, {\mathcal {U}}^{p}_{a}(t_{n+1}))\Big ]+ l_{4},\\&{}^{{\mathfrak {c}}}{\mathcal {D}}^{\gamma }{\mathcal {I}}_{d}(t_{j+1})= \frac{{\textbf{h}}^{\gamma }}{\Gamma (\gamma +2)}\Big [\sum ^{n}_{j=0} d_{j,n+1} {\mathfrak {L}}_{5}(t_{j}, {\mathcal {U}}_{a}(t_{j}))+ {\mathfrak {L}}_{5}(t_{n+1}, {\mathcal {U}}^{p}_{a}(t_{n+1}))\Big ]+ l_{5},\\&{}^{{\mathfrak {c}}}{\mathcal {D}}^{\gamma }{\mathcal {H}}(t_{j+1})= \frac{{\textbf{h}}^{\gamma }}{\Gamma (\gamma +2)}\Big [\sum ^{n}_{j=0} d_{j,n+1} {\mathfrak {L}}_{6}(t_{j}, {\mathcal {U}}_{a}(t_{j}))+ {\mathfrak {L}}_{6}(t_{n+1}, {\mathcal {U}}^{p}_{a}(t_{n+1}))\Big ]+ l_{6},\\&{}^{{\mathfrak {c}}}{\mathcal {D}}^{\gamma }{\mathcal {R}}(t_{j+1})= \frac{{\textbf{h}}^{\gamma }}{\Gamma (\gamma +2)}\Big [\sum ^{n}_{j=0} d_{j,n+1} {\mathfrak {L}}_{7}(t_{j}, {\mathcal {U}}_{a}(t_{j}))+ {\mathfrak {L}}_{7}(t_{n+1}, {\mathcal {U}}^{p}_{a}(t_{n+1}))\Big ]+ l_{7}. \end{aligned} \end{aligned}$$

## Numerical simulation and discussion

**Figure 2:**
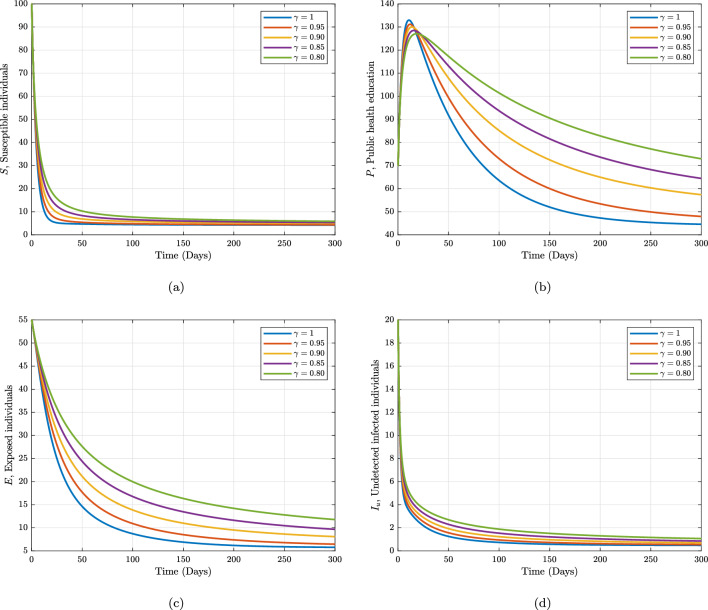
Numerical trajectory of Marburg transmission under Caputo fractional operator.

**Figure 3:**
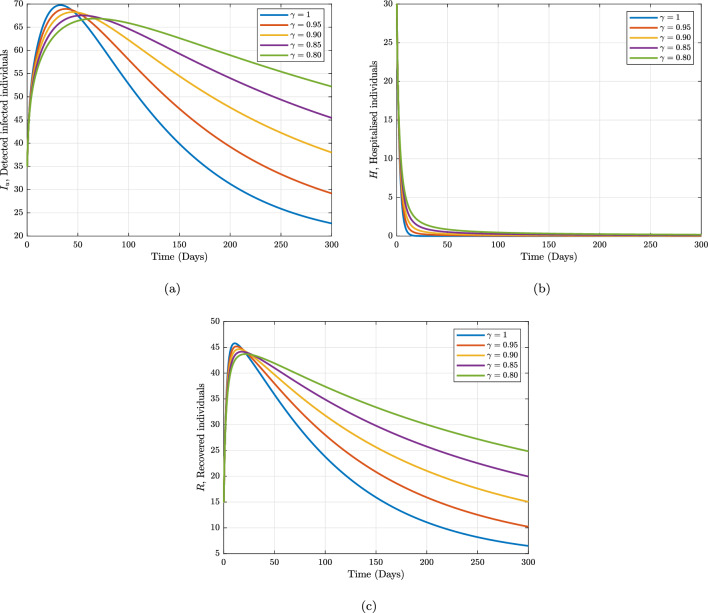
Numerical trajectory of Marburg transmission under Caputo fractional operator.

For the purpose of validating our created iterative scheme, we present our numerical simulation in this part. For this, we start with initial values for each compartment of our proposed model (1); ${\mathcal {S}}= 100; {\mathcal {P}}= 70; {\mathcal {E}}= 55; {\mathcal {I}}_{u}= 20; {\mathcal {I}}_{d}= 35; {\mathcal {H}}=30; {\mathcal {R}}= 15.$ We have employed Adam-Bashforth-Moulton scheme to obtain numerical solution to the system. We compare the effects of various fractional order values with a step size 0.2 throughout the time range [0,300] against the suitable parameter values listed in Table 1. Figures 2 and 3 represent the numerical simulation results for the individuals. It is clear that the outcomes and the changes of the fractional-order $\gamma$ fit well, which indicate that the method is effective, thus when the operator $\gamma$ is varied the dynamism of each state variable has the same trend. However, their values are slightly different. For example, when the fractional order $\gamma$ is reduced from 1 in Fig. 2a and b show the differences between susceptible and susceptible individuals received health education. These figures demonstrate that the number of susceptible educated individuals increases over time until it achieves a carrying capacity while the number of susceptible individuals decreases over time as more and more people contract the infection. In Fig. 2c, the exposed population initially increase with fast speed corresponding to small fractional order then became slow. Similar to Fig. 2d, the undetected infected people. This graph shows a rapid decrease in the initial 50 days and later on it goes towards stability. In Fig. 3a there is sharp increase for all values of fractional operator $\gamma$ due to the high transmissibility of Marburg virus of the disease advocated according to WHO. In Fig. 3b, the hospitalised population initially increase up with fast speed corresponding to small fractional order then became slow and later on it goes towards stability. During this time the recovery population also achieve their maximum peak in the initial 40 days as shown in Fig. 3c. These figures demonstrate that the Caputo derivative generates global dynamics of the suggested model, where lower orders reach stability more quickly than integer orders.

**Figure 4:**
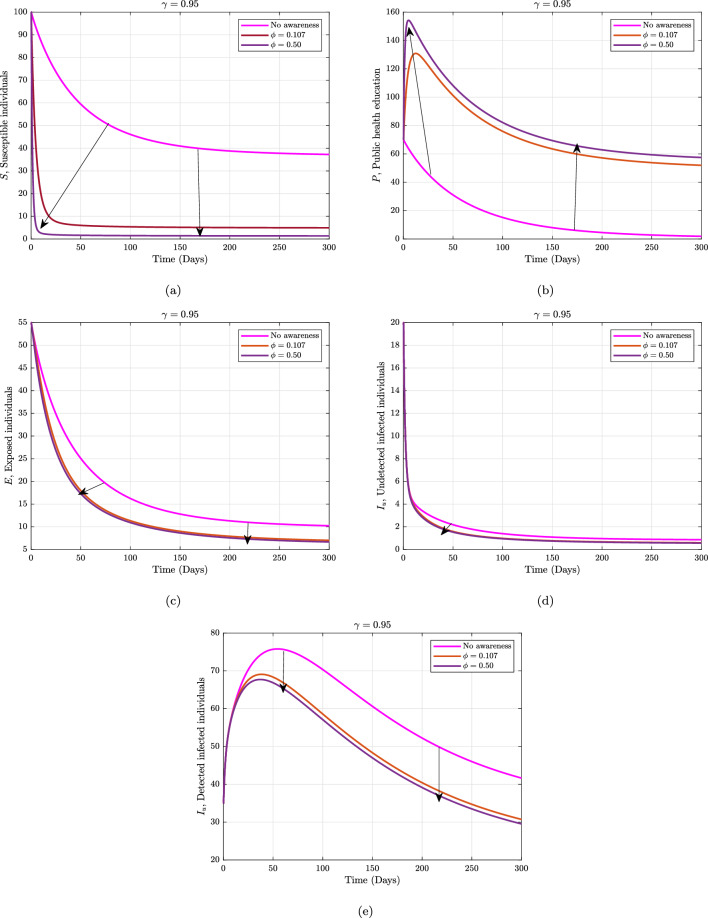
Numerical trajectory when one varying awareness rate against fractional operator $\alpha =0.95$.

**Figure 5:**
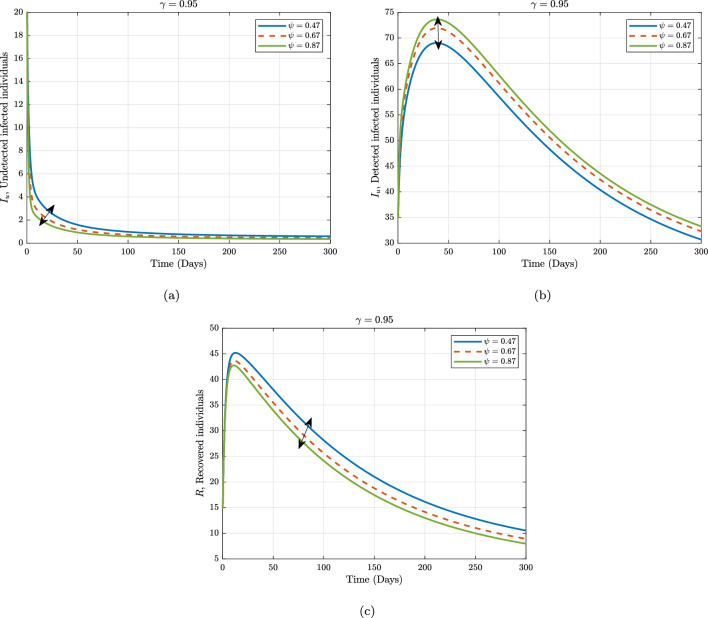
Numerical trajectory when one varying detection rate against fractional operator $\alpha =0.95$.

Furthermore, considering the contributions of some of the sensitive parameters in our proposed model, we maintain the fractional operator to be fixed at $\gamma =0.95$ and varied the information dissemination and the rate of detecting unknown Marburg virus infected individuals in Figs. 4 and 5, respectively. Figure 4 depicts the impact of the various values of information dissemination or awareness rate on the dynamics of $S, P, E, I_{u},$ and $I_{d}$. In general, this figure reflects that when information dissemination increases, the number of $S, E, I_{u},$ and $I_{d}$ decreases rapidly. This indicates how educating susceptible people about their health can help to stop the spread of the Marburg virus. As a result, in order to stop and limit the spread of the Marburg virus, public policymakers must concentrate on enhancing the value of information dissemination. Fig. 5 depicts the impact of the various values of detecting unknown Marburg virus infected individuals on the dynamics of $I_{u}, I_{d}$ and *R*. We noticed that dynamics has significant impact on our proposed model. These results give public policymakers a note on how and where resources allocate are needed in order to prevent and control Marburg virus spread.

## Conclusion

In recent years, numerous deadly diseases have appeared in many countries around the globe. If the limitations of established methodologies, ideas, and procedures are updated, questioned, and amended in response to contemporary scientific findings and the emergence of unforeseen physical phenomena, the dynamics of infectious diseases can be better understood and even predicted. We have comprehensively analyzed a new deterministic mathematical model for Marburg virus in a homogeneously mixing human population under Caputo fractional order derivative in this paper. We have investigated the qualitative aspect of the spread of the Marburg virus by analysing the positiveness, boundedness, equilibrium point, and fundamental reproductive number $\mathfrak {R_0}$. The Banach contraction mapping principle is used to show the system existence and uniqueness analysis. The fractional controlling system of equations underwent a stability study using the Hyers-Ulam-type stability criteria. Using the Adam-Bashforth-Moulton scheme, numerical trajectories are constructed to test the effectiveness of the suggested fractional-order model. We looked at the impact of some critical parameters. Based on the trajectories, we hypothesized that the memory index or fractional order can be use by the public health policymakers to comprehend and foresee the dynamics of the transmission of the Marburg virus. It is also seen that if information dissemination and availability of resources to detect infected individuals can reduce the spread of Marburg virus infection. This study consider no real data which is our limitation. Despite this limitation, this model provides a good description of ongoing Marburg outbreak. The model looks into the use of public health education, which is a crucial component of disease control in the modern era.

## Data Availability

The data sets used and/or analyzed during the current study available from the corresponding author on reasonable request.
