# Author Correction: A nonlinear fractional epidemic model for the Marburg virus transmission with public health education

**DOI:** 10.1038/s41598-023-47506-w

**Published:** 2023-12-15

**Authors:** Emmanuel Addai, Adejimi Adeniji, Mercy Ngungu, Godfred Kuffuor Tawiah, Edmore Marinda, Joshua Kiddy K. Asamoah, Muhammad Altaf Khan

**Affiliations:** 1 College of Computer and Information Science, University of Arkansas at Little Rock, Little Rock, Arkansas State 72204 USA; 2 Department of Mathematics and Statistics, Tshwane University of Technology, Pretoria, South Africa; 3 Human Sciences Research Council (HSRC), Pretoria, South Africa; 4 College of Biomedical Engineering, Taiyuan University of Technology, Shanxi, Taiyuan 030024 China; 5 Department of Mathematics, Kwame Nkrumah University of Science and Technology, Kumasi, Ghana; 6 Faculty of Natural and Agricultural Sciences, University of the Free State, Bloemfontein, South Africa

Correction to: *Scientific Reports* 10.1038/s41598-023-46127-7, published online 07 November 2023

In the original version of this Article, the affiliations of Emmanuel Addai were incorrectly given as ‘Department of Mathematics, Taiyuan University of Technology, Shanxi, Taiyuan, 030024, China’, ‘College of Biomedical Engineering, Taiyuan University of Technology, Shanxi, Taiyuan, 030024, China’, and ‘College of Computer and Information Science, University of Arkansas, Fayetteville, Arkansas State, United States’. The correct affiliation is:

College of Computer and Information Science, University of Arkansas at Little Rock, Little Rock, Arkansas State, 72204, USA

In addition, the spelling of the given name of the author Muhammad Altaf Khan was incorrectly given as ‘Mohammed Altaf’.

The original Article has been corrected.

